# Synergistic Interaction of 5-HT_1B_ and 5-HT_2B_ Receptors in Cytoplasmic Ca^2+^ Regulation in Human Umbilical Vein Endothelial Cells: Possible Involvement in Pathologies

**DOI:** 10.3390/ijms241813833

**Published:** 2023-09-08

**Authors:** Elena Yu. Rybakova, Piotr P. Avdonin, Sergei K. Trufanov, Nikolay V. Goncharov, Pavel V. Avdonin

**Affiliations:** 1 Koltsov Institute of Developmental Biology, Russian Academy of Sciences, Moscow 119334, Russia; alenka3107@mail.ru (E.Y.R.); ppavdonin@gmail.com (P.P.A.); gad.91@inbox.ru (S.K.T.); 2 Sechenov Institute of Evolutionary Physiology and Biochemistry, Russian Academy of Sciences, Saint Petersburg 194223, Russia; ngoncharov@gmail.com

**Keywords:** endothelial cells, serotonin, 5-HT1B and 5-HT2B receptors, calcium, pathology, hypertension, COVID-19

## Abstract

The aim of this work was to explore the involvement of 5-HT_1B_ and 5-HT_2B_ receptors (5-HT_1B_R and 5-HT_2B_R) in the regulation of free cytoplasmic calcium concentration ([Ca^2+^]_i_) in human umbilical vein endothelial cells (HUVEC). We have shown by quantitative PCR analysis, that 5-HT_1B_R and 5-HT_2B_R mRNAs levels are almost equal in HUVEC. Immunofluorescent staining demonstrated, that 5-HT_1B_R and 5-HT_2B_R are expressed both in plasma membrane and inside the cells. Intracellular 5-HT_1B_R are localized mainly in the nuclear region, whereas 5-HT_2B_R receptors are almost evenly distributed in HUVEC. 5-HT, 5-HT_1B_R agonist CGS12066B, or 5-HT_2B_R agonist BW723C86 added to HUVEC caused a slight increase in [Ca^2+^]_i_, which was much lower than that of histamine, ATP, or SFLLRN, an agonist of protease-activated receptors (PAR1). However, activation of 5-HT_1B_R with CGS12066B followed by activation of 5-HT_2B_R with BW723C86 manifested a synergism of response, since several-fold higher rise in [Ca^2+^]_i_ occurred. CGS12066B caused more than a 5-fold increase in [Ca^2+^]_i_ rise in HUVEC in response to 5-HT. This 5-HT induced [Ca^2+^]_i_ rise was abolished by 5-HT_2B_R antagonist RS127445, indicating that extracellular 5-HT acts through 5-HT_2B_R. Synergistic [Ca^2+^]_i_ rise in response to activation of 5-HT1BR and 5-HT_2B_R persisted in a calcium-free medium. It was suppressed by the phospholipase C inhibitor U73122 and was not inhibited by the ryanodine and NAADP receptors antagonists dantrolene and NED-19. [Ca^2+^]_i_ measurements in single cells demonstrated that activation of 5-HT_2B_R alone by BW723C86 caused single asynchronous [Ca^2+^]_i_ oscillations in 19.8 ± 4.2% (*n* = 3) of HUVEC that occur with a long delay (66.1 ± 4.3 s, *n* = 71). On the contrary, histamine causes a simultaneous and almost immediate increase in [Ca^2+^]_i_ in all the cells. Pre-activation of 5-HT_1B_R by CGS12066B led to a 3–4 fold increase in the number of HUVEC responding to BW723C86, to synchronization of their responses with a delay shortening, and to the bursts of [Ca^2+^]_i_ oscillations in addition to single oscillations. In conclusion, to get a full rise of [Ca^2+^]_i_ in HUVEC in response to 5-HT, simultaneous activation of 5-HT_1B_R and 5-HT_2B_R is required. 5-HT causes an increase in [Ca^2+^]_i_ via 5-HT_2B_R while 5-HT_1B_R could be activated by the membrane-permeable agonist CGS12066B. We hypothesized that CGS12066B acts via intracellular 5-HT_1B_R inaccessible to extracellular 5-HT. Intracellular 5-HT_1B_R might be activated by 5-HT which could be accumulated in EC under certain pathological conditions.

## 1. Introduction

The 5-hydroxytryptamine (5-HT) was first isolated by Vittorio Erspamer from enterochromaffin cells of the intestine as a low-molecular substance that causes smooth muscle contraction [1]. Later, 5-HT was isolated and crystallized from bovine blood serum, and its chemical structure has been established [2]. It caused the contraction of an isolated blood vessel, the ear artery of a rabbit, so it was named serotonin. About 90% of the body’s 5-HT is produced in the digestive tract in the enterochromaffin cells [3]. In the brain, 5-HT is synthesized in neurons in raphe nuclei of the brain stem [4]. The 5-HT is also produced in the pulmonary endothelial [5] and neuroendocrine [6] cells, skin Merkel cells [7], and taste buds [8]. The action of 5-HT is mediated by seven types of 5-HT receptors (5-HTR), six of which are G-protein-coupled receptors (5-HT_1,2,4,5,6,7_R), and one type (5-HT_3_R) is a cationic channel [9]. Within these types, there are subtypes of receptors, and the total number of genes coding 5-HTR is 18 (https://www.guidetopharmacology.org, accessed on 20 May 2023). All subtypes of 5-HT receptors are expressed in the mammalian brain. Their functions in the central nervous system and involvement in neurological pathologies have been intensively studied for decades [9]. Despite the discovery of 5-HT as a substance that causes vasoconstriction, the 5-HT receptors in blood vessels received less attention [10].

The majority of 5-HT, which enters the bloodstream, is synthesized by the enterochromaffin cells of the human small intestinal mucosa and is transported by platelets. The average plasma concentration of 5-HT is very low; according to various estimates, it ranges from 1 to 100 nM [10]. However, in some pathological conditions, it can increase greatly [10,11]. In particular, patients with COVID-19 had significantly increased plasma 5-HT and 5-hydroxyindoleacetic acid (5-HIAA) levels compared with healthy donors [12]. The 5-HT and 5-HIAA plasma concentrations increased with higher severity of symptoms. It has been hypothesized that 5-HT is a putative mediator linking systemic manifestations, such as pulmonary, intestinal, and cardiac, which characterize the severe course of COVID-19 in individuals with diabetes and obesity [13]. Altered concentrations of circulating 5-HT are implicated in several other pathologic conditions, including atherosclerosis and primary and secondary hypertension [14].

Violations of 5-HT metabolism in pathologies are accompanied by changes in the functional activity of 5-HT receptors. Normally, 5-HT contracts blood vessels via 5-HT_2A_R and 5-HT_1B_R localized in smooth muscle cells [10,15]. However, in the hypertension [16,17] and diabetes, 5-HT_2B_R is also involved in the 5-HT-induced vasoconstriction [18]. Elevated vasoconstriction in deoxycorticosterone acetate (DOCA)-salt and N(omega)-nitro-L-arginine (L-NAME) hypertensive rats correlates with the elevation of 5-HT_2B_R expression [16,17]. On the contrary, enhanced contraction of arteries from diabetics to 5-HT via 5-HT_2B_R in a mouse model of type II diabetes mellitus is not accompanied by an increase in their expressions [18]. Oxidative stress and the generation of excessive reactive oxygen species (ROS) are known to be associated with the development of metabolic diseases, including diabetes [19]. It can be suggested that, in this case, ROS affects the sensitivity of cells to serotonin. We have demonstrated that in smooth muscle cells (SMCs) from rat aorta, there are functionally inactive, “silent” 5-HT_2B_R, which, under conditions of artificially induced oxidative stress, acquire the ability to stimulate a rise in [Ca^2+^]_i_ level in SMCs [20]. Under the influence of oxidative stress, the isolated rat mesenteric artery and aorta became able to contract in response to the activation of 5-HT_2B_R by its agonist BW723C86.

Thus, the above data indicate the existence of a link between the state of 5-HT_2B_R and the development of several vascular pathologies. For a more complete understanding of the mechanisms of pathogenesis of these diseases, a detailed study of the regulation of the [Ca^2+^]_i_ level by 5-HT_2B_R and other 5-HT receptors in vascular cells is required. Endothelial cells (EC) are the first target of 5-HT in blood vessels and are in the frontline protecting vascular wall from elevated concentrations of 5-HT in plasma. However, little is known about the effect of 5-HT on [Ca^2+^]_i_ in EC and the role of the individual receptors in this process. Vascular EC express 5-HT_2B_R coupled with G_q/11_ protein and phospholipase C, 5-HT_1B_R coupled with Gi protein and adenylyl cyclase (AC) inhibition, and 5-HT_4_ receptors coupled with Gs protein and AC activation [10]. Information on the effect of 5-HT on calcium metabolism in EC is fragmentary and somewhat contradictory. According to [21], 5-HT at a concentration of up to 100 μM does not induce [Ca^2+^]_i_ elevation in HUVEC. The authors suggested that 5-HT-induced secretion of von Willebrand factor in HUVEC is mediated by a decrease in cAMP level due to activation of 5-HT_1B_R. On the other hand, it has been shown that in EC from human coronary arteries, activation of 5-HT_1B_R and 5-HT_2B_R causes an increase in [Ca^2+^]_i_ [22]. Ullmer et al. [23] demonstrated that the 5-HT_2B_R agonist BW723C86 causes an increase in [Ca^2+^]_i_ in human pulmonary artery EC by activating ryanodine-sensitive reticulum channels, while 5-HT_1B_R is not involved in the regulation of calcium metabolism [23]. According to our data, both 5-HT_1B_R and 5-HT_2B_R in the presence of hydrogen peroxide markedly elevate [Ca^2+^]_i_ in HUVEC [24]. On the contrary, H_2_O_2_ attenuates histamine-induced [Ca^2+^]_i_ rise in these cells [25].

The aim of this work was to further explore the regulation of [Ca^2+^]_i_ via 5-HT_1B_R and 5-HT_2B_R in HUVEC and to compare the effects of their agonists with the effects of histamine and agonists of other receptors that cause an increase in [Ca^2+^]_i_ in EC. CGS12066B and BW723C86 were used to stimulate 5-HT_1B_R and 5-HT_2B_R, respectively. Since both 5-HT_1B_R and 5-HT_2B_R might be involved in the action of 5-HT on calcium metabolism in HUVEC, we investigated what would be the changes in [Ca^2+^]_i_ when they are simultaneously activated by their selective agonists. Surprisingly, it has been found that their action on [Ca^2+^]_i_ in HUVEC exhibits a strong synergy. Registration of the responses of individual cells has demonstrated that simultaneous stimulation of 5-HT_1B_R and 5-HT_2B_R results in an increase in the number of the cells in which [Ca^2+^]_i_ rise occurs and initiates the bursts of [Ca^2+^]_i_ oscillations in addition to single oscillations. We have also demonstrated that 5-HT induces very weak [Ca^2+^]_i_ rise in HUVEC via 5-HT_2B_R, and 5-HT_1B_R agonist CGS12066B greatly enhances the 5-HT effect.

## 2. Results

**Figure 1:**
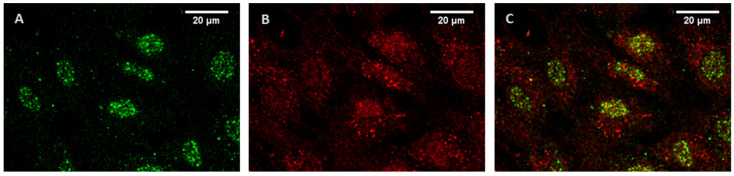
Immunofluorescent staining of 5-HT_1B_R (**A**) and 5-HT_2B_R (**B**) in HUVEC. (**C**)—overlay of (**A**) and (**B**).

Previous PCR analyses demonstrated the expression of mRNA of 5-HT_1B_R and 5-HT_2B_R in HUVEC [26]. Using quantitative PCR, we also showed the presence of mRNA encoding 5-HT_1B_R and 5-HT_2B_R in these cells (Supplementary Figure S1). The amounts of 5-HT_1B_R and 5-HT_2B_R mRNAs were nearly equal. In HUVEC, a low level of expression of mRNA encoding of 5-HT_2C_R was revealed. The amount of 5-HT_2C_R mRNA was less than 0.5% of 5-HT_1B_R or 5-HT_2B_R mRNA. HT_2A_R mRNA is absent in HUVEC. The presence of 5-HT_1B_R and 5-HT_2B_R proteins in HUVEC was demonstrated by Western blot hybridization (Supplementary Figure S2) and immunofluorescent staining. To determine whether 5-HT_1B_R and 5-HT_2B_R are localized not only in the plasma membrane but also within HUVEC, the cells were stained with antibodies in the absence and presence of Triton X-100. It was found that 5-HT_1B_R and 5-HT_2B_R were expressed both on the cell surface (Supplementary Figure S3) and inside the cells (Supplementary Figure S4). We determined intracellular localization of 5-HT_1B_R and 5-HT_2B_R in HUVEC at higher resolution. As shown in Figure 1, the majority of 5-HT_1B_R is localized in the nuclear region, while 5-HT_2B_R is distributed relatively evenly in the cells.

**Figure 2:**
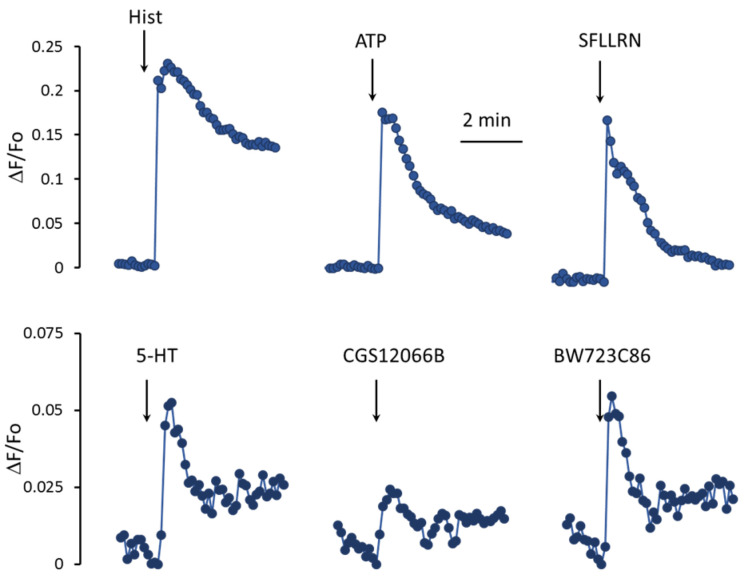
An increase in [Ca^2+^]_i_ in HUVEC in response to histamine, ATP, SFLLRN, 5-HT, and 5HT_1B_R and 5HT_2B_R agonists CGS12066B and BW723C86. The increase in [Ca^2+^]_i_ was estimated as the relative increase in CalciumGreen fluorescence, where Fo is the fluorescence registered immediately before the addition of receptor agonists to the cells, and ΔF is the difference between Fo and fluorescence at certain time points. The concentrations of histamine, ATP, 5-HT, BW723C86, CGS12066B, and SFLLRN were 10, 100, 10, 30, 50 μM, and 10 μg/mL, respectively. Fluorescence was measured at intervals of 8 s.

In EC, the metabolism of calcium ions is activated by histamine, ATP, thrombin, and a number of other factors [27,28]. As shown in Figure 2, histamine, an agonist of protease-activated receptors type 1 SFLLRN, and ATP cause a rapid increase in mean [Ca^2+^]_i_ levels in a population of HUVEC. Unlike histamine and other agonists, 5-HT acts much weaker. In response to 100 μM 5-HT, the increase in [Ca^2+^]_i_ is three–five times lower. In addition, the rate of rise in the [Ca^2+^]_i_ level in the cell population in response to 5-HT is also significantly slower than in response to histamine, SFLLRN, and ATP. To elucidate the role of 5-HT_1B_R and 5-HT_2B_R in the regulation of [Ca^2+^]_i_ in HUVEC, we used their agonists, CGS12066B [29] and BW723C86 [30], respectively. The 5-HT_1B_R agonist CGS12066B caused a weaker increase in mean [Ca^2+^]_i_ levels in the HUVEC compared to 5-HT. The 5-HT_2B_R agonist BW723C86 at a concentration of 50 µM caused an increase in [Ca^2+^]_i_ comparable to that of 5-HT by magnitude and rate.

**Figure 3:**
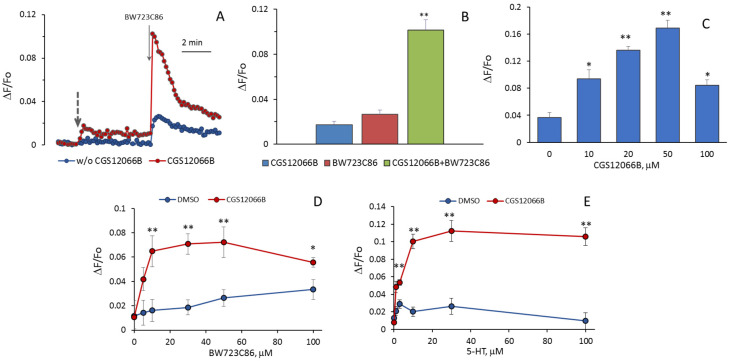
Potentiation by CGS12066B of [Ca^2+^]_i_ rise in HUVEC in response to BW723C86 and 5-HT. (**A**) Kinetics of [Ca^2+^]_i_ rise in response to BW723C86 (30 μM) in the presence or absence of CGS12066 (50 μM). Dashed line arrow indicates the addition of CGS12066B or vehicle (0.1% DMSO in PSS). (**B**) The values of calcium signals of HUVEC in response to CGS12066B, BW723C86, or both. (**C**) Effects of different concentrations of CGS12066B on [Ca^2+^]_i_ rise induced by BW723C86 (30 μM). [Ca^2+^]_i_ rises in response to different concentrations of BW723C86 (**D**) or 5-HT (**E**) after preincubation without or with CGS12066B (50 μM). * *p* < 0.05; ** *p* < 0.01 when compared to CGS12066B and BW723C86 in (**B**) and to the corresponding controls in (**C**–**E**).

It could be expected that 5-HT increases [Ca^2+^]_i_ as a result of activation of 5-HT_1B_R and 5-HT_2B_R. We assumed that an additive effect would be observed when agonists of these receptors were added to HUVEC, but it turned out that CGS12066B and BW723C86 have a synergistic effect on [Ca^2+^]_i_. After preincubation with 50 µM CGS12066B, the rise in [Ca^2+^]_i_ in HUVEC in response to 30 µM BW723C86 increased three–five times (Figure 3A,B). In a separate experiment, the cells were preincubated with different concentrations of CGS12066B for 5 min, and after that, we determined the response to 30 μM BW723C86 (Figure 3C). CGS12066B potentiated the calcium response to BW723C86 starting from a concentration of 10 μM; the maximum enhancement occurred at a CGS12066B concentration of about 50 μM, and this effect was weakened at 100 μM. We have studied whether and how 5-HT_1B_R activation would affect the concentration dependence of the increase in [Ca^2+^]_i_ in response to BW723C86 and 5-HT. After preincubation with 50 μM CGS12066B, BW723C86 causes an increase in [Ca^2+^]_i_ already at 5 μM, and at 10 μM, the effect of BW723C86 reaches its maximum (Figure 3D). In the presence of CGS12066B [Ca^2+^]_i_, elevation occurs at 1–3 μM of 5-HT and further increases at 10–100 μM (Figure 3E). Without CGS12066B, the effect of 5-HT on [Ca^2+^]_i_ was either rather small (Figure 2) or almost absent (Figure 3E).

**Figure 4:**
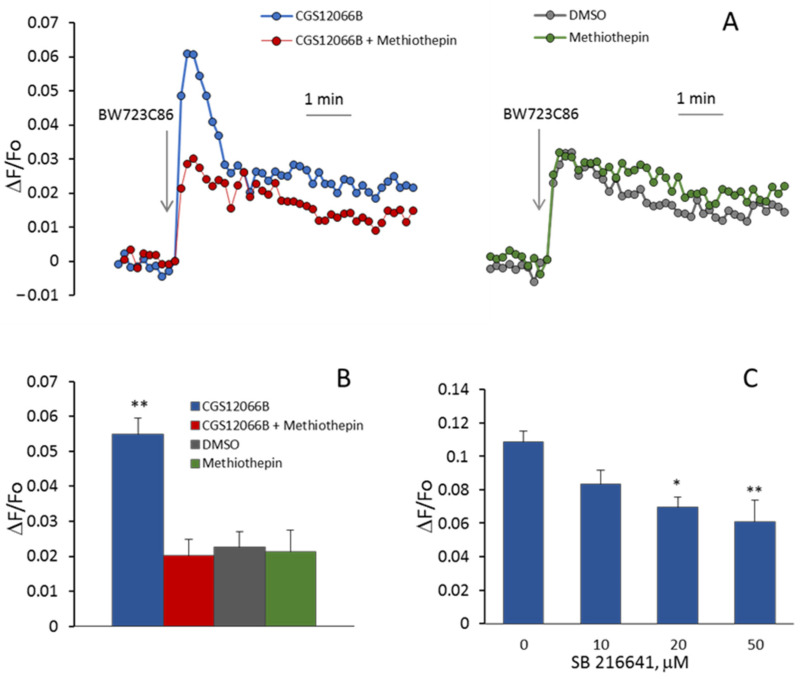
Effects of methiothepin (**A**,**B**) and SB216641 (**C**) on BW723C86-induced [Ca^2+^]_i_ elevation in HUVEC in the presence of CGS12066B. The cells were incubated with 10 μM methiothepin or various concentrations of SB216641 for 5 min and then, with 50 μM CGS12066B for 5 min. After that, 30 μM BW723C86 was added. DMSO dissolved in PSS at appropriate concentrations was added to the cells as a vehicle control. Methiothepin did not suppress [Ca^2+^]_i_ elevation induced by BW723C86 without CGS12066B (**A**,**B**). * *p* < 0.05, ** *p* < 0.01 compared to control without SB 216641.

**Figure 5:**
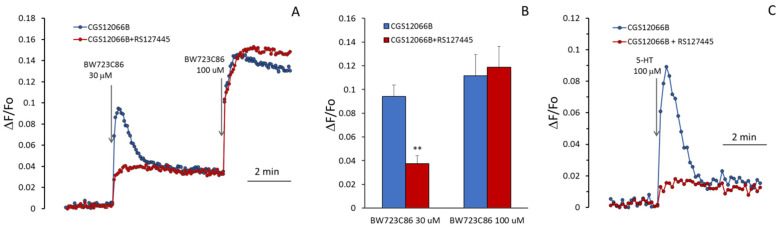
Effect of RS127445 on [Ca^2+^]_i_ rise in HUVEC in response to BW723C86 and 5-HT. The cells were incubated for 5 min in the absence or presence of 10 μM RS127445, then, in the presence of 50 μM CGS12066B for 5 min; after that, 30 and 100 μM BW723C86 or 100 μM 5-HT were added to the cells. (**A**) Kinetics [Ca^2+^]_i_ changes and (**B**) values of the maximum increase in [Ca^2+^]_i_ (means ± SEM, *n* = 6, ** *p* < 0.01 compared to control without RS127445) after the addition of BW723C86. (**C**) [Ca^2+^]_i_ changes in response to 5-HT with or without RS127445.

In additional experiments, we determined the effects of 5-HT_1B_R and 5-HT_2B_R antagonists on the [Ca^2+^]_i_ rise induced by CGS12066B and BW723C86 in HUVEC. As shown in Figure 4A,B, the 5-HT_1B_R antagonist methiothepin [31] at a concentration of 10 μM abolished potentiation by CGS12066B of BW723C86-induced Ca^2+^ mobilization. According to [32], methiothepin also binds to 5-HT_2B_R. In our experiments, methiothepin did not suppress the rise in [Ca^2+^]_i_ induced by BW723C86 in the absence of CGS12066B. This indicates that methiothepin affects 5-HT_2B_R in HUVEC. We explored the effect of another 5-HT_1B_R antagonist, SB216641 [33]. SB216641 at concentrations of 10 to 50 μM was added 5 min before CGS12066B (50 μM). Then, [Ca^2+^]_i_ responses to BW723C86 (30 μM) were recorded. As shown in Figure 4C, SB216641 caused a concentration-dependent decrease in [Ca^2+^]_i_ rise. The 5-HT_2B_R antagonist RS127445 [34] suppressed calcium response to 30 µM BW723C86 (Figure 5A,B), but the blocking effect of RS127445 disappeared after a subsequent increase in the concentration of BW723C86 to 100 µM. This indicates a competition between these two compounds for binding to the 5-HT_2B_R. CGS12066B strongly increases the response to 5-HT (Figure 5C), which is suppressed by the 5-HT_2B_R antagonist RS127445.

**Figure 6:**
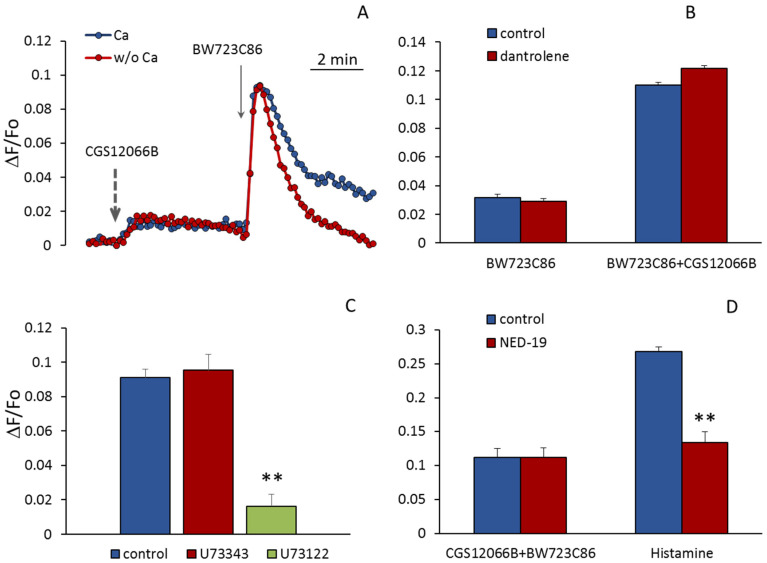
Increase in [Ca^2+^]_i_ in response to CGS12066B and BW723C86 in the absence or presence of calcium ions in the extracellular medium (**A**) and the effect of dantrolene (**B**), U73122 (**C**), and NED-19 (**D**) on [Ca^2+^]_i_ rise induced by CGS12066B and BW723C86. As a control, the influence of NED-19 on the rise in [Ca^2+^]_i_ by histamine is shown (**D**). The curves on (**A**) are obtained by averaging 10 independent measurements. In (**B**–**D**), each value is a mean ± SEM, *n* = 10. ** *p* < 0.01 compared to control and U73343. In (**D**), the histamine-induced [Ca^2+^]_i_ in the absence and presence of NED-19 is demonstrated. In (**A**) kinetics of [Ca^2+^]_i_, changes were registered either with 0.2 mM EGTA and without Ca^2+^ or with 1 mM CaCl_2_ in the medium. Concentrations of dantrolene and NED-19 were 50 μM, and concentrations of U73343 and U73122—2 μM.

According to Ullmer et al. [23], upon activation of 5-HT_2B_R in human pulmonary artery EC, an increase in [Ca^2+^]_i_ occurs as a result of the release of Ca^2+^ from the reticulum through ryanodine-sensitive channels (RyR). It could be assumed that CGS12066 potentiates the EC response to BW723C86 due to the influx of Ca^2+^ ions from the outside and triggering Ca^2+^-induced Ca^2+^-release (CICR). However, as shown in Figure 6A, the removal of calcium ions from the extracellular environment does not reduce the rise in [Ca^2+^]_i_ induced by BW723C86 in the presence of added CGS12066B. Dependence on extracellular calcium manifests itself in the second slow phase of the response to BW723C86, which indicates an inward current of Ca^2+^, apparently by the mechanism of store-operated calcium entry. It has been shown that EC of the human mesenteric artery and immortalized EA.hy926 endothelial-derived cells express RyR3, whereas RyR1 and RyR2 were not detected in these cells [35]. RyR3 is known to be inhibited by dantrolene [36]. We used this compound to find out if RyRs in HUVEC are involved in the potentiation of the response to BW723C86 by CGS12066B. In our experiments, dantrolene at a concentration of 50 μM did not reduce the rise in [Ca^2+^]_i_ in HUVEC in response to BW723C86 alone and together with CGS12066B (Figure 6B). In contrast, inhibition of phospholipase C activity by U73122 almost completely abolished the calcium signal from 5-HT_2B_R (Figure 6C). We hypothesized that potentiation of the BW723C886-induced [Ca^2+^]_i_ increase by CGS12066B could be due to the release of calcium ions from acidic endolysosomal vesicles via two-pore channels (TPC) activated by NAADP. One of the functions of these channels is to trigger global calcium release by recruiting CICR channels at lysosomal–endoplasmic reticulum (ER) junctions [37]. The structural analog of NAADP substance NED-19 is a blocker of these channels [38]. NAADP-activated channels have been shown to be involved in histamine-induced Ca increase in HUVEC [39]. To find out if these channels are activated under the influence of CGS12066B and, thus, potentiate the action of BW723C86, we used NED-19 and revealed that it did not affect the rise in [Ca^2+^]_i_ under the influence of CGS12066B and BW723C86, although attenuated histamine-induced [Ca^2+^]_i_ elevation (Figure 6D).

**Figure 7:**
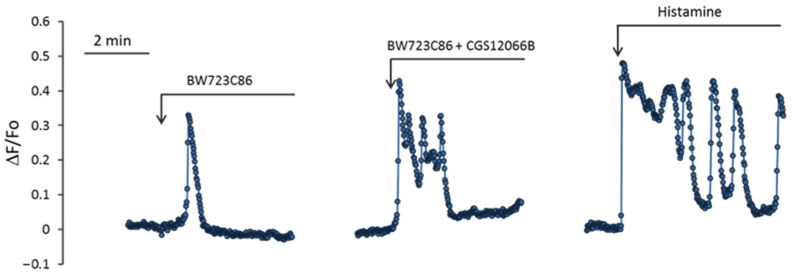
Kinetics of [Ca^2+^]_i_ changes in single HUVEC in response to BW723C86, to BW723C86 in the presence of CGS12066B, and to histamine. Characteristic curves of calcium responses are presented. The concentrations of BW723C86, CGS12066B, and histamine were, respectively, 30, 50, and 1 μM.

For a better understanding of how the calcium signal of HUVEC is enhanced with simultaneous activation of 5-HT_1B_R and 5-HT_2B_R, we registered changes in [Ca^2+^]_i_ in single cells. Fluorescence measurement was performed using a Leica DMI6000 microscope in the second–fourth passages of HUVEC cultured in 24-well plates. It has been previously shown that in response to the activation of membrane receptors in single EC [Ca^2+^]_i_, changes occur in the form of oscillations [40]. Figure 7 shows characteristic oscillations when BW723C86 alone was added to HUVEC, when BW723C86 was added to HUVEC 5 min after CGS12066B, and when histamine was added to the cells. The fluorescence of CalciumGreen was recorded entirely from the whole cell. Processing of fluorescent signals from each cell in the field of view was carried out.

**Figure 8:**
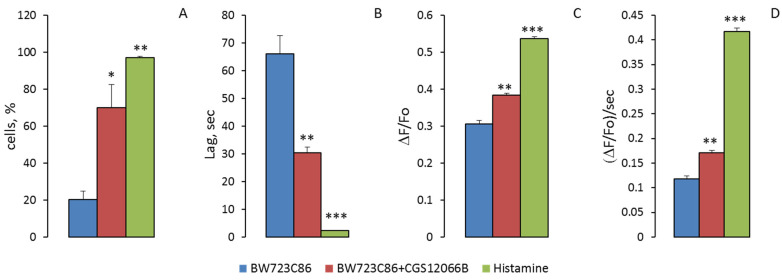
Proportion of HUVEC in which BW723C86, BW723C86, together with CGS12066B or histamine induce [Ca^2+^]_i_ oscillations (**A**) and characteristics of [Ca^2+^]_i_ oscillations—delay before oscillation onset (**B**), magnitude of oscillation (**C**), and maximum rate of [Ca^2+^]_i_ increase during the development of the oscillation (**D**). Means ± SEM are given. (**A**) presents data from three independent measurements for each value. In (**B**,**C**,**D**) the mean values from the measurements in 71, 227, and 292 cells for BW723C86, BW723C86 + CGS12066B, and histamine, respectively, are presented. * *p* < 0.05 when compared to BW723C86 alone in (**A**); in (**B**,**C**,**D**) ** *p* < 0.01 when compared to BW723C86 alone, and *** *p* < 0.001 when compared to BW723C86 and BW723C86 with CGS12066B.

Theoretically observed potentiation of the response to BW723C86 in a population of HUVEC can occur for several reasons: as a result of an increase in the number of responding cells; synchronization of [Ca^2+^]_i_; rises in individual cells in the population; an increase in maximal [Ca^2+^]_i_; rise at oscillation; and the appearance of secondary oscillations. It can be seen from the graphs in Figure 7 that the responses of individual cells to BW712C86 in the absence and presence of CGS12066B sharply differ. When only BW723C86 was added to HUVEC, they responded with single oscillations. The relative number of cells in which [Ca^2+^]_i_ oscillations occurred was 19.8 ± 4.2% (*n* = 3) (Figure 8A). Measurements of [Ca^2+^]_i_ were performed in three wells with the average number of HUVEC in the field of view 116 ± 3 (mean ± SEM). Within 5 min after the addition of BW723C86, secondary single oscillations of [Ca^2+^]_i_ occurred in 2.3 ± 0.5% (*n* = 3) of cells. After preincubation with CGS12066B, the cells responded to BW723C86 not only with single oscillations but also with bursts of oscillations. Responses to BW723C86 occurred in 69.7 ± 12.4% (*n* = 3) cells. Secondary single oscillations or bursts of oscillations in response to BW723C86 added after CGS12066B occurred in 39.7 ± 13.2% (*n* = 3) of HUVEC. Calcium responses of HUVEC to BW723C86 after preincubation with CGS12066B resembled those to histamine (Figure 7). However, histamine calcium response was much stronger—it caused bursts of oscillation and secondary oscillations in 98 ± 1.4% (*n* = 3) of HUVEC.

The responses of single cells to BW723C86 in the form of oscillations occurred with a much longer delay than responses to BW723C86 added after CGS12066B or responses to histamine (Figure 7). The average times (means ± SEM) to reach the peak of the oscillation after adding BW723C86 and BW723C86 in the presence of CGS12066B or histamine were 66.1 ± 4.3 s (*n* = 71; 95% CI for mean 53.0 to 79.1 s), 30.5 ± 1.9 s (*n* = 227; 95% CI for mean 26.8 to 34.2 s), and 2.31 ± 0.04 s (*n* = 292; 95% CI for means 2.24 to 2.38 s), respectively. This indicates that CGS12066B partially synchronizes the calcium responses of a single HUVEC.

We determined the parameters of [Ca^2+^]_i_ oscillations arising in response to VW723C86, to BW723C86 in the presence of CGS12066B, and to histamine. The values of maximal [Ca^2+^]_i_ rise at the peaks of oscillations (max ΔF/Fo), and the maximum rate of [Ca^2+^]_i_ increase (ΔF/Fo per s) during oscillations were calculated (Figure 8). The mean values of max ΔF/Fo (mean ± SEM) under the action of BW723C86 and BW723C86 in the presence of CGS12066B or histamine were 0.306 ± 0.009 (*n* = 71; 95% CI for the mean 0.2878 to 0.3248), 0.414 ± 0.0678 (*n* = 227; 95% CI for the mean 0.3719 to 0.3951), and 0.5369 ± 0.0054 (*n* = 292; 95% CI for the mean 0.5262 to 0.5475). The maximum rates of [Ca^2+^]_i_ rise (ΔF/Fo per second) were 0.118 ± 0.0061 s^−1^ for BW723C86 (*n* = 71; 95% CI for the mean 0.1058 to 0.1302), 0.171 ± 0.004 s^−1^ (*n* = 227; 95% CI for the mean 0.1616 to 0.1802) for BW723C86 with CGS12066B, and for histamine 0.4165 ± 0.0078 s^−1^ (*n* = 292; 95% CI for means 0.4010 to 0.4320). The presented data indicate that the increase in [Ca^2+^]_i_ under the combined action of CGS12066B and BW723C86 occurs as a result of an increase in the number of reacting cells, synchronization of their responses, the appearance of bursts of oscillations and repeated oscillations, and an increase in the rate of [Ca^2+^]_i_ rise.

## 3. Discussion

In this work, we have demonstrated that 5-HT_1B_R and 5-HT_2B_R in HUVEC are localized both in the plasma membrane and inside the cells. Intracellular 5-HT_1B_R is located predominantly in the nuclear region, while 5-HT_2B_R is distributed rather evenly in the cells. Expression in the nuclear membrane has been shown for different G-protein-coupled receptors (GPCRs) [41,42]. Various nuclear receptors activate G-proteins and stimulate the formation of second messengers (cAMP, IP3, etc.). With regard to 5-HT and other classical neurotransmitters, the idea of the existence of intracellular receptors functioning in cells at the pre-nervous stages of development was proposed many years ago [43]. It has been shown that endogenous 5-HT is formed in cells already at the stage of cleavage divisions [44]. An argument in favor of the existence of intracellular 5-HT receptors was the ability of lipophilic 5-HT derivatives to influence cleavage divisions and the absence of such an effect in hydrophilic 5-HT derivatives [45].

We have found that the activation of 5-HT_1B_R by CGS12066B by itself causes very slight changes in [Ca^2+^]_i_ but increases the calcium signal from 5-HT_2B_R in HUVEC several-fold. The potentiating effect of CGS12066B begins to manifest itself at concentrations of up to 10 µM, and at 50 µM, the effect of CGS12066B reaches its maximum. According to previously published data [46], CGS12066A activates the binding of [^35^S]GTPγS to rat striatal membranes with an EC50 of 1.2 µM and has a maximum effect at concentrations of 20 µM. These values are close to concentrations of CGS12066B, potentiating the calcium response to BW723C86 in HUVEC. Of note, the IC50 value for CGS12066B at the 5HT_1B_R recognition site is 51 nM, as determined using the binding of [3H]5HT in the presence of 1 μM spiperone [29]. A possible explanation for the quantitative discrepancy in the data on CGS12066B binding and on its functional effects may be the difference in the affinity for the agonist of the inactive G-protein uncoupled and the active G-protein coupled receptors.

To activate 5-HT_2B_R, its agonist BW723C86 was used. It was demonstrated that this ligand could bind to all three types of 5-HT_2_ receptors [32]. As it was shown in [26] and in our work, 5-HT_2A_Rs are not expressed in HUVEC. We have also demonstrated that the content of 5-HT_2C_R mRNA in HUVEC is very low. Involvement of 5-HT_2C_R [Ca^2+^]_i_ rise in response to BW723C86 or 5-HT can be excluded since it is suppressed by 5-HT_2B_R antagonist RS127445. We hypothesize that CGS12066B enhances the response to BW723C86 and 5-HT by activating intracellular 5-HT_1B_R. CGS12066B is a lipophilic compound capable of diffusing through the plasma membrane and crossing the blood–brain barrier [29]. We suggest that the lack of 5-HT action through 5-HT_1B_R can be explained by the fact that 5-HT, unlike CGS12066B, cannot freely pass through the plasma membrane and bind to 5-HT_1B_R inside the cell.

The next question concerns the mechanism by which 5-HT_2B_R activates the rise in [Ca^2+^]_i_ and how the response to BW723C86 is potentiated. It has been shown previously that in EC from the human pulmonary artery, this compound via 5-HT_2B_R stimulates calcium release from intracellular stores through a pathway, which involves activation of ryanodine receptors and is independent of PI-hydrolysis [23]. In contrast, in our experiments, blocking the activity of phospholipase C in HUVEC by 2 μM U73122 almost completely suppresses the rise in [Ca^2+^]_i_ in response to BW723C86, while the RyR inhibitor dantrolene does not affect it. This suggests that 5-HT_2B_R activates the release of calcium ions through InsP3-activated channels (InsP3R). InsP3R is a calcium ion-permeable channel formed by four protein subunits that, similar to RyR, are additionally activated by calcium ions [47]. In the cytoplasmic part of the InsP3R monomers, there are high-affinity Ca^2+^-binding sites that modulate the activity of the channel. According to [48], the activation of these channels occurs as follows: the binding of inositol-1,4,5-trisphosphate to InsP3R causes the formation of clusters of single channels, and elevation of [Ca^2+^]_i_ above the basal level increases the probability of channel open state, reduces the time of the closed state, and ensures their simultaneous opening in the cluster. Thus, there is an increase both in the intensity and duration of the current of calcium ions through the cluster formed by InsP3Rs. We suggest that the potentiation of the calcium signal from 5-HT_2B_R under the action of the 5-HT_1B_R agonist CGS12066B can be mediated by Ca^2+^-induced activation of InsP3R. This hypothesis is consistent with our data on an increase in the [Ca^2+^]_i_ rise rate and peak height of BW723C86-induced calcium oscillations in single cells under the influence of CGS12066B. At the first stage of the calcium response to BW723C86 added after CGS1206B, [Ca^2+^]_i_ elevates due to its mobilization from intracellular depots since the maximal rise and the rate of [Ca^2+^]_i_ elevation does not depend on the presence of calcium ions in the extracellular medium. After emptying the intracellular depots, the entry of calcium ions from the outside by the SOCE mechanism, the refilling of the reticulum, and repeated oscillations appear. We assume that CGS12066B locally elevates [Ca^2+^]_i_ and, in this way, potentiates InsP3-dependent Ca^2+^ release from the reticulum induced by BW723C86. The presence of a lag phase before the onset of Ca^2+^ oscillations in HUVEC in response to activation of 5-HT_2B_R can apparently be explained by a slow increase in the local [Ca^2+^]_i_ to the threshold level required to open InsP_3_-sensitive channels. CGS12066B causes a slight increase in [Ca^2+^]_i_ in HUVEC. The intracellular source from which calcium ions are released in response to CGS12066B has not been established. We have shown that NED-19, a blocker of NAADP-stimulated two-pore channels, does not reduce the effect of CGS12066B, suggesting that acid endolysosomal vesicles are not involved in this process. The question of how the synergism is realized with the simultaneous activation of 5-HT_1B_R and 5-HT_2B_R needs further investigation.

An increase in [Ca^2+^]_i_ is a trigger of many physiological and pathological processes. It has been shown that, normally, 5-HT through 5-HT_2B_R regulates differentiation and proliferation during development as well as cardiac structure and function in adults [49]. Changes in the functional activity and expression of 5-HT_2B_R can cause disturbances in the regulation by 5-HT of normal physiological processes and contribute to the development of pathologies. Increased activation of 5-HT_2B_R by the fenfluramines due to the off-target effect cause valvulopathy [50]. According to [51], the initial steps of mitral valve remodeling involved the mobilization of bone marrow-derived endothelial progenitor cells by 5-HT_2B_R stimulation. The 5-HT is implicated in the growth of various malignant cell types [52]. There is evidence of the involvement of 5-HT_1B_R and 5-HT_2B_R in these pathological processes [53,54]. Primary pulmonary hypertension is associated with a substantial increase in 5-HT_2B_R expression in pulmonary arteries [55]. At the same time, in pulmonary hypertension, the synthesis of 5-HT in EC increases [5], which can cause activation of intracellular 5HT_1B_R. As a result, 5-HT, as we assume, may induce a sharp increase in [Ca^2+^]_i_ in the EC, leading to their damage. The risk of pulmonary hypertension in humans is increased under the action of dexfenfluramine, which is an active metabolite and an agonist of 5-HT_2B_R. Bloodworth et al. [56] demonstrated that bone marrow-derived proangiogenic cells contribute to experimental pulmonary hypertension in a 5-HT_2B_R signaling-dependent manner. It is worth noting that SB216641, a 5-HT_1B_R antagonist, prevented the development of pulmonary hypertension in a ROS-dependent manner [57]. Dysregulation of intracellular calcium ions is a factor causing hypertension and related pathologies. In cultured smooth muscle cells from rat aorta, the Ca^2+^-mobilizing activity of 5-HT_2B_R is elevated under the action of the compounds that cause the generation of ROS [20]. We have previously shown that ROS also affects the serotonergic regulation of calcium metabolism in HUVEC: the rise in [Ca^2+^]_i_ caused by the activation of 5-HT_1B_R, and 5-HT_2B_R is significantly increased by the addition of H_2_O_2_ [24], and, conversely, hydrogen peroxide inhibited the rise in [Ca^2+^]_i_ induced by histamine [25]. In the present work, we have found another feature in serotoninergic calcium regulation in these cells—the synergistic interaction of 5-HT_1B_R and 5-HT_2B_R upon activation of the rise in [Ca^2+^]_i_. In the vascular bed, a potent vasoconstrictor and an activator of smooth muscle cell proliferation endothelin-1 (ET-1) is produced by EC and released preferentially to the basal side of endothelium [58]. One can speculate that an excessive rise in [Ca^2+^]_i_ leads to endothelial hypersecretion of ET-1, and this may be an additional factor in the pathogenesis of hypertension. The increase in the calcium response of EC to 5-HT via 5-HT_2B_R upon activation of 5-HT_1B_R should apparently be taken into account as a potential cause of 5-HT undesirable effects.

## 4. Materials and Methods

### 4.1. Reagents

CGS12066B, BW723C86, RS127445, and SB216649 were from Tocris Bioscience (Bristol, UK); 5-HT, histamine, ATP, SFLLRN, U73122, U73343, and dantrolene were from Sigma-Aldrich (St. Louis, MO, USA); CalciumGreen/AM was from Thermo Fischer Scientific (Waltham, MA, USA). The stock solutions of CGS12066B, BW723C86, RS127445, SB216649, dantrolene, U73122, and U73343 were prepared in Dimethylsulfoxide (DMSO). Before being added to the cells, they were dissolved to the required concentrations in a physiological salt solution (PSS). DMSO at appropriate concentrations was used as a vehicle control. PSS was added as a vehicle control for 5-HT, ATP, histamine, and SFLLRN.

### 4.2. Cell Culture

HUVEC were isolated, according to [59]. The cells were grown in plastic flasks pre-coated with gelatin, using M199 medium with Earl’s salts and 20 mM HEPES containing 20% fetal calf serum (Sigma-Aldrich), 300 µg/mL endothelial growth supplement, isolated from rabbit brain, 100 µg/mL heparin, and gentamicin. We used the cells on early passages (2–5). Accutase^®^ was applied for passaging the cells (Sigma-Aldrich).

### 4.3. Immunofluorescence

To determine the expression of 5-HT_1B_R and 5-HT_2B_R in the plasma membrane and inside HUVEC, the cells grown in a 48-well plate were fixed in 4% paraformaldehyde in 0.01 M phosphate-buffered saline (PBS) for 20 min. Fixed cells were incubated in PBS with 1% BSA without Triton X-100 or in the presence of 0.1% Triton X-100 for 20 min at 4 °C. The non-permeabilized cells were incubated overnight at +4 °C with HTR1B rabbit pAB (ABclonal, Woburn, MA, USA, A18285) or with HTR2B rabbit pAB (ABclonal, A5670), each diluted 1:100. Then, the cells were stained with donkey anti-rabbit Alexa Fluor™ 647 (Thermo Fischer Scientific, A-31573) diluted 1:500. Permeabilized with 0.1% Triton X-100 HUVEC were incubated with goat anti-5-HT1BR (MyBioSource, San Diego, CA, USA MBS420311) at a concentration of 1 μg/mL in PBS and HTR2B rabbit pAB (ABclonal, A5670), diluted 1:100. The cells were stained with a mixture of chicken anti-goat Alexa Fluor™ 488 (Thermo Fischer Scientific, A-21467) and donkey anti-rabbit Alexa Fluor™ 647 (Thermo Fischer Scientific, A-31573). The nuclei were stained with 10 μg/mL Hoechst 33342 (Biotium Inc., Fremont, CA, USA, #40044). The preparations of intact and permeabilized HUVEC were analyzed using a Leica THUNDER fluorescent microscope (Leica, Wetzlar, Germany), HC PL FLUOTAR L 20×/0.40 lens, and Quad Filter Block DFT51010.

In order to obtain a higher resolution of 5-HT_1B_R and 5-HT_2B_R, HUVEC were grown on a glass coverslip. The cells were fixed in 4% paraformaldehyde in 0.01 M phosphate-buffered saline (PBS) for 20 min. After washing three times in PBS, the cells were permeabilized in a 0.1% solution of Triton X-100 in PBS with 1% bovine serum albumin (BSA) for 10 min. After that, the cells were incubated for 24 h at +4 °C in a mixture of primary antibodies: goat anti-5-HT_1B_R (MyBioSource, San Diego, CA, USA MBS420311) and rabbit anti-5-HT_2B_R (BIOSSUSA, Woburn, MA, USA, bs-1892R) at a concentration of 1 μg/mL in PBS with 1% BSA, washed in PBS and stained with a mixture of secondary antibodies: chicken anti-goat Alexa Fluor™ 488 (Thermo Fischer Scientific, A-21467) and donkey anti-Rabbit Alexa Fluor™ 594 (Thermo Fischer Scientific, A-21207) diluted 1:500 12 h at +4 °C. The preparations were analyzed using a Leica DMI 6000 fluorescent microscope (Leica, Germany) using an HCX PL APO CS 40.0 × 1.25 OIL UV lens (Leica, Germany), diode illuminators with wavelengths of 488 and 594 nm and fluorescent filter cubes L5 ET, and TX2 ET (Leica, Germany), respectively.

### 4.4. Western Blot

HUVEC grown in a 25 cm^2^ flask were lysed in RIPA-buffer (Thermo Fisher Scientific, Waltham, MA, USA, #89901) containing protease inhibitor cocktail (Thermo Fischer Scientific, #78430) with 5 mM EDTA. The proteins (35 μg per lane) were separated on 10% PAAG in Tris-Glycine buffer (25 mM TRIS-base, 250 mM Glycine, 0.1% SDS) and electroblotted onto Immun-Blot PVDF membrane (BioRad Laboratories Inc., Hercules, CA, USA, #162-0177). After transfer, the membrane was blocked with 5% non-fat dry milk in TTBS (20 mM tris-Cl, pH 7.4, 0.5 M NaCl, 0.1% Tween 20) for 2 h at room temperature, cut into strips, and left to incubate with 5-HT1BR goat pAb (MyBioSource, MBS420311) at 1:150 dilution or HTR2B Rabbit pAb (Abclonal, A5670) at 1:1000 dilution in blocking buffer overnight at 4 °C. Then, the membrane strips were washed three times and incubated with secondary antibodies: HRP-conjugated goat anti-rabbit (BioRad #170-6515) or donkey anti-goat (R&D Systems, Minneapolis, MN, USA, #HAF109) at 1:1000 dilution for 1 h at room temperature. After incubation, the strips were washed three times and developed with Clarity MaxTM Western ECL Substrate #1705062. Luminescence was detected at the C-Digit reader manufactured by LI-COR Biosciences (Omaha, NE, USA).

### 4.5. Measurement of Free Cytoplasmic Calcium Concentration in HUVEC

HUVEC grown in 96-well plates were loaded with 1 µM CalciumGreen/AM dissolved with 0.02% Pluronic F-127 in M199 during 1 h at 37 °C in a CO2 incubator (New Brunswick Scientific, Edison, NJ, USA). Measurement of [Ca^2+^]_i_ was performed in physiological salt solution (PSS) containing NaCl (145 mM), KCl (5 mM), MgCl_2_ (1 mM), CaCl_2_ (1 mM), HEPES (5 mM), and D-glucose (10 mM) at pH 7.4. Fluorescence was registered at 485 nm (excitation) and 530 nm (emission) at 25 °C using a Synergy 4 Microplate Reader (BioTek, Winooski, Vermont, USA). The changes in [Ca^2+^]_i_ in HUVEC are presented as the ratio (F − Fo)/Fo or ΔF/Fo, where Fo is the basal fluorescence, and F is the fluorescence at time points during recording. Each curve in the graph is a superposition of five to ten curves recorded independently.

Measurement of [Ca^2+^]_i_ in single cells was carried out using a Leica DMI 6000 fluorescent microscope (Leica, Germany) using an HCX PL FLUOTAR L 20.0 × 0.40 DRY objective (Leica, Germany), diode illuminator with wavelengths of 480/40 nm and fluorescent filter L5 ET (Leica, Germany). HUVEC were grown in 24-well plates and loaded with 1 µM CalciumGreen/AM, as described above. Fluorescence was recorded from the area of 334 × 447 microns in the center of the well. Fluorescence measurements were taken at 1 s intervals. The resulting images were converted to tiff files and analyzed using the CellProfiler program [60] free at http://cellprofiler.org/releases (accessed on 20 May 2023). Cells were isolated as separate objects, in each of which the kinetics of fluorescence changes were determined. The results are plotted as the ratio (F−Fo)/Fo or ΔF/Fo, where Fo is the fluorescence of a single cell before the addition of BW723C86 and F is the fluorescence at each time point during the recording. The maximum rate of increase in [Ca^2+^]_i_ during the oscillation was determined from the increase in ΔF/Fo during one second during the development of the oscillation using Microsoft Excel 2010.

### 4.6. Measurement of mRNA Coding of 5-HT_1B_R, 5-HT_2A_R, 5-HT_2B_R, 5-HT_2C_R, and hEF1A in HUVEC

Total RNA was isolated from HUVEC using the Aurum™ Total RNA Mini Kit (Bio-Rad Laboratories, Hercules, CA, USA). The purity and concentration of RNA were assessed by absorption at 260 and 280 nm using a NanoDrop 8000 spectrophotometer (Thermo Fisher Scientific, Waltham, MA, USA). For reverse transcription, a High-Capacity RNA-to-cDNA Kit (Thermo Fisher Scientific, Waltham, MA, USA) was used. Real-time PCR was performed on a 7500 Real-Time PCR System (Thermo Fisher Scientific, USA) with reagent mixture qPCRmix-HS LowROX (Eurogen, Moscow, Russia). Ct values for 5-HTRs genes and Ct values for human elongation factor 1A (EF1A) were determined in the same cDNA preparation. The relative amounts of mRNAs for 5-HTRs were calculated according to the following equation: fold gene expression = 2^−(∆∆Ct)^ [61]. The content of 5-HT2BR mRNA was the highest; therefore, it was taken as a calibrator.

The following TaqMan probes and primers specific to human 5-HT_1B_R, 5-HT_2A_R, 5-HT_2B_R, and 5-HT_2C_R were used.

5-HT_1B_R Sense CTTCTGGCGTCAGGCTAAGG

5-HT_1B_R Antisense GAGTAGACCGTGTAGAGGATGTG

5-HT_1B_R Probe FAM-TCACCACGCATTCCGACACCTCCT-BHQ1

5-HT_2A_R Sense ATCTCGCTGGACCGCTACG

5-HT_2A_R Anti-sense CAACTCCCCTCCTTAAAGACCTTC

5-HT_2A_RProbe FAM-CCAGAATCCCATCCACCACAGCCGC-BHQ1

5-HT_2B_R Sense TGCTGACAAAGGAACGTTTTGG

5-HT_2B_RAntisense TTAGGCGTTGAGGTGGCTTG

5-HT_2B_R Probe FAM-TGCTCTTTGGCTCACTGGCTGCCT-BHQ1

5-HT_2C_R Sense CGCCGACAAGCTTTGATGTTAC

5-HT_2C_R Antisense CTTGCAGCACTTCAGGAAATCC

5-HT_2C_R Probe FAM-CCACACCGAGGAACCGCCTGGACT-BHQ1

hEF1A Sense CCATGTGTGTTGAGAGCTTCTCA

hEF1A Antisense CTTGTCCACTGCTTTGATGACAC

hEF1A Probe FAM-CTATCCACCTTTGGGTCGCTTTGCT-BHQ1

### 4.7. Statistics

Data are presented as mean ± SEM of 5 to 10 measurements. In each case, at least three independent experiments were performed with different cell preparations in which similar results were obtained. Statistical significance was calculated using the unpaired Student’s *t*-test (Microsoft Excel 2010) and one-way ANOVA, according to the Student–Newman–Keuls test (MedCalc, Version 14.8.1, Ostend, Belgium).

## 5. Conclusions

HUVEC express 5-HT_1B_ and 5-HT_2B_ receptors. We have demonstrated that 5-HT_1B_R and 5-HT_2B_R are localized both in the plasma membrane and inside the cells. The majority of intracellular 5-HT_1B_R are localized in the nuclear region, while 5-HT_2B_R are distributed relatively evenly in the cell. For the full manifestation of their Ca^2+^-mobilizing activity, simultaneous stimulation of both 5-HT_2B_R and 5-HT_1B_R is required. The 5-HT causes an increase in [Ca^2+^]_i_ in HUVEC via 5-HT_2B_R when 5-HT_1B_R are activated by the membrane-permeable agonist CGS12066B. We hypothesize that the potentiation of [Ca^2+^]_i_ rise by CGS12066B is mediated by intracellular 5-HT_1B_R, which are inaccessible to 5-HT added from the outside. We assume that intracellular 5-HT_1B_R are activated by 5-HT when it is accumulated in EC under certain pathological conditions.

## Data Availability

The data presented in this study are available on request from the corresponding author.

## Supplementary Materials

The supporting information can be downloaded at: https://www.mdpi.com/article/10.3390/ijms241813833/s1.
