# Supplementary material for: Synergistic Interaction of 5-HT1B and 5-HT2B Receptors in Cytoplasmic Ca2+ Regulation in Human Umbilical Vein Endothelial Cells: Possible Involvement in Pathologies
Source: Int J Mol Sci. 2023 Sep 8;24(18):13833. doi: 10.3390/ijms241813833 (PMC10530667; doi:10.3390/ijms241813833)
Supplement: Supplementary file 1 [file ijms-24-13833-s001.zip › ijms-2464470-supplementary.pdf]

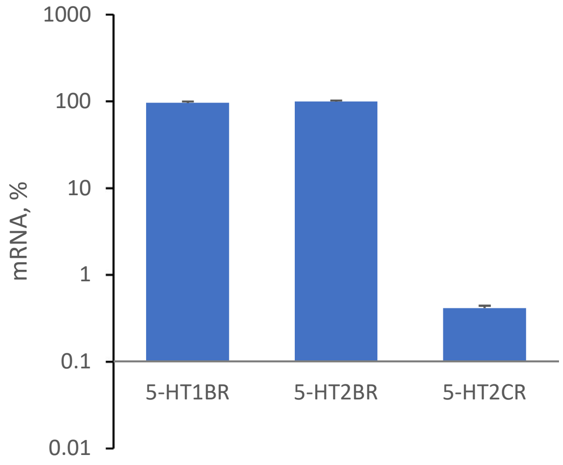

Supplementary Figure S1. Expression of 5-HT_{1B}R, 5-HT_{2B}R and 5-HT_{2C}R mRNA in HUVECs. To calculate relative contents of mRNAs the following equation was used: fold gene expression = $2^{-\Delta\Delta Ct}$. Human EF1A was a standard. The Ct values for EF1A, 5-HT1BR, 5-HT_{2B}R, and 5-HT_{2C}R were 19.984±0.016, 27.992±0.009, 27.942±0.026, and 35.856±0.121 respectively. 5-HT_{2A}R mRNA was not detected. The mRNA content of 5-HT2B receptors was taken as 100%. The means ± SEM of 4 measurements are presented.

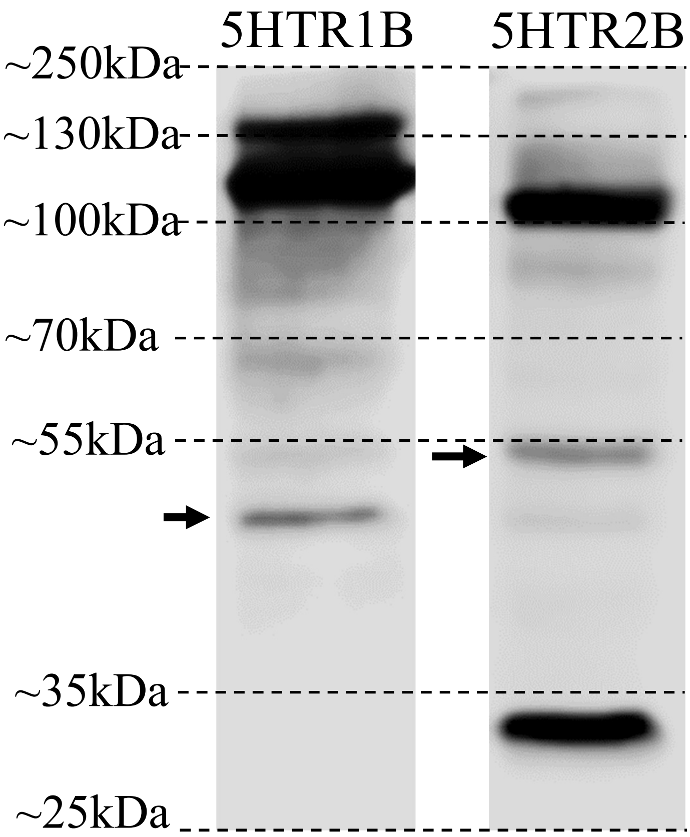

Supplementary Figure S2. Western blot analysis of extracts of HUVECs using anti-5-HT1BR goat pAb (MyBioSource, MBS420311) at 1:150 dilution and anti-5-HTR2B Rabbit pAb (Abclonal, A5670) at 1:1000 dilution.

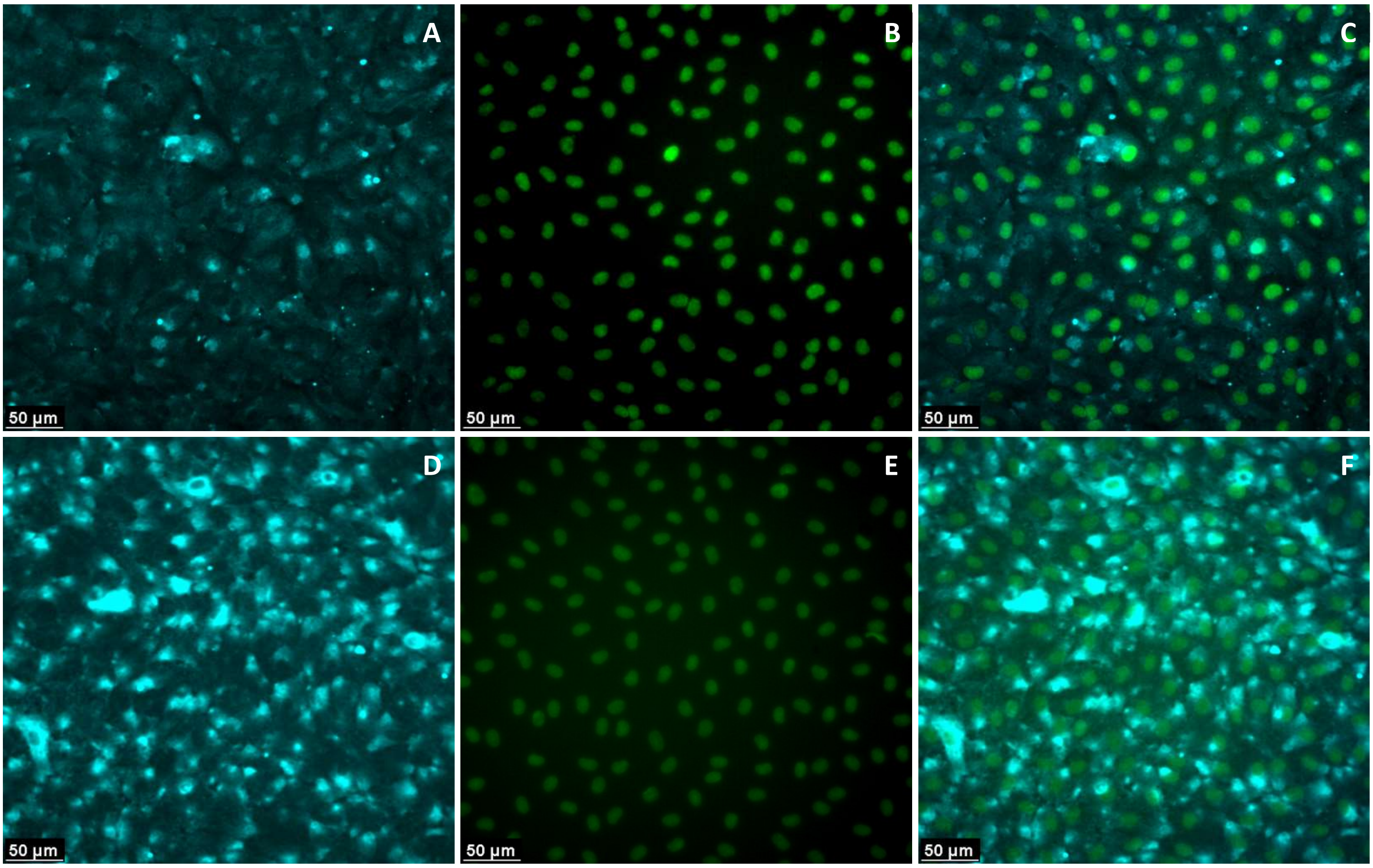

Supplementary Figure S3. Immufluorescent staining of 5-HT$_{1B}$R (A) and 5-HT$_{2B}$R (D) in unpermeabilized HUVECs. Cell nuclei were stained with Hoechst 33342 (B,E). (C) – overlay of (A) and (B), (F) – overlay of (D) and (E). HT$_{1B}$R were stained with rabbit pAB (ABclonal, A18285) targeted to amino acids 1-390 of human HT$_{1B}$R. HT$_{2B}$R were stained with rabbit pAB (ABclonal, A5670) targeted to amino acids 233-330 of human HT$_{2B}$R.

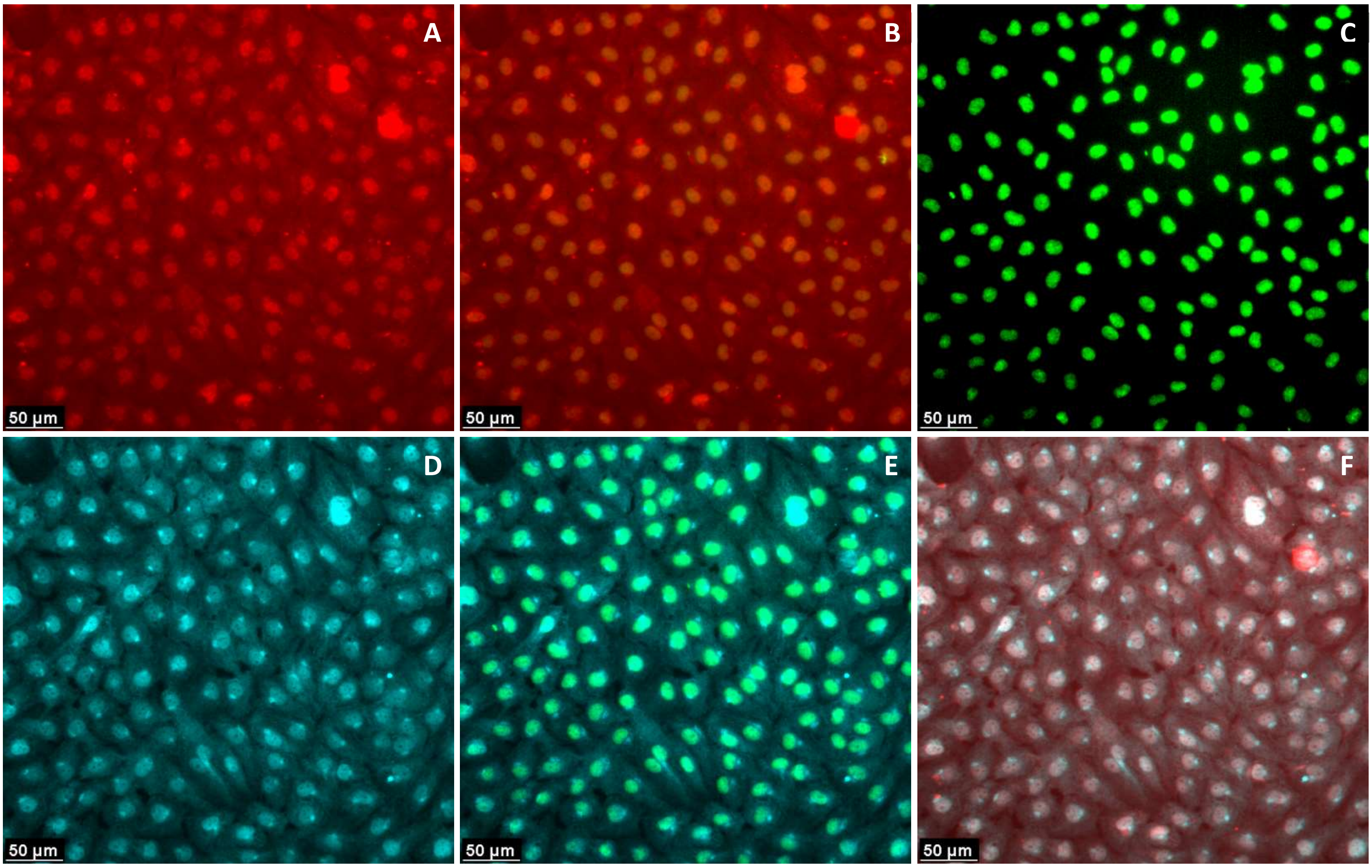

Supplementary Figure S4. Immufluorescent staining of 5-$HT_{1B}R$ (A) and 5-$HT_{2B}R$ (D) in HUVECs permeabilized with 0.1% Triton X-100. Cell nuclei were stained with Hoechst 33342 (C). (B) – overlay of (A) and (C), (E) – overlay of (D) and (C), (F) – overlay of (A) and (D). 5-$HT_{1B}R$ were stained with goat anti-5-HT1BR (MyBioSource, MBS420311) targeted to amino acids 372-382. 5-$HT_{2B}R$ were stained with rabbit pAB (ABclonal, A5670) targeted to amino acids 233-330.
